# Pain Related Channels Are Differentially Expressed in Neuronal and Non-Neuronal Cells of Glabrous Skin of Fabry Knockout Male Mice

**DOI:** 10.1371/journal.pone.0108641

**Published:** 2014-10-22

**Authors:** Jarmila Lakomá, Roberto Rimondini, Vincenzo Donadio, Rocco Liguori, Marco Caprini

**Affiliations:** 1 Laboratory of Human and General Physiology, Department of Pharmacy and Biotechnology (FaBiT), University of Bologna, Bologna, Italy; 2 Department of Medical and Clinical Sciences (DIMEC), University of Bologna, Bologna, Italy; 3 IRCCS Institute of Neurological Sciences, AUSL Bologna, Bologna, Italy; 4 Department of Biomedical and Neuromotor Sciences (DIBINEM), University of Bologna, Bologna, Italy; University of Pittsburgh School of Medicine, United States of America

## Abstract

Fabry disease (FD) is one of the X-linked lysosomal storage disorders caused by deficient functioning of the alpha-galactosidase A (*α*-GalA) enzyme. The *α*-GalA deficiency leads to multi-systemic clinical manifestations caused by the preferential accumulation of globotriaosylceramide in the endothelium and vascular smooth muscles. A hallmark symptom of FD patients is peripheral pain that appears in the early stage of the disease. Pain in FD patients is a peripheral small-fiber idiopathic neuropathy, with intra-epidermal fiber density and integrity being used for diagnosing FD in humans. However, the molecular correlates underlying pain sensation in FD remain elusive. Here, we have employed the *α*-GalA gene KO mouse as a model of FD in rodents to investigate molecular changes in their peripheral nervous system that may account for their algesic symptoms. The *α*-GalA null mice display neuropathic pain as evidenced by thermal hyperalgesia and mechanical allodynia, with histological analyses showing alterations in cutaneous innervation. Additionally, KO mice showed a decreased and scattered pattern of neuronal terminations consistent with the reduction in neuronal terminations in skin biopsies of patients with small fiber neuropathies. At the molecular level KO animals showed an increase in the expression of TRPV1 and Nav1.8, and a decrease in the expression of TRPM8. Notably, these alterations are observed in young animals. Taken together, our findings imply that the *α*-GalA KO mouse is a good model in which to study the peripheral small fiber neuropathy exhibited by FD patients, and provides molecular evidence for a hyperexcitability of small nociceptors in FD.

## Introduction

Neuropathic pain is a very common and disabling symptom in numerous small fiber neuropathies (SFN), involving at the same time sensory and emotional experiences. One of such SFNs, Fabry disease (FD) is a hereditary X-linked metabolic storage disorder due to insufficient amounts or a complete lack of the lysosomal enzyme *α*-galactosidase A (*α*-GalA). The loss of *α*-*GalA* activity leads to an abnormal accumulation of globotriaosylceramide (Gb3) in lysosomes and other cellular components of different tissues and cell types, affecting the cell function. FD is a multi-system disorder, with an extremely wide range of physical signs and symptoms related to the gender of the affected patient. The most characteristic neurological manifestation in hemizygous males is a latching painful neuropathyaffecting mainly the feet, legs and hands [1].

Differently, heterozygous female patients with FD exhibit heterogeneous phenotypic variations of the clinical features, ranging from asymptomatic to severe symptoms due to random X-chromosomal inactivation [2]–[4].

Pain perception is carried out through small diameter nerve fibers to the central nervous system and is also associated with FD. Specifically, it has been demonstrated that FD patients show reduced intraepidermal nerve fiber density and impaired thermal perception [5]–[7]. The underlying molecular and functional mechanisms of pain in SFN patients with Fabry disease are still not completely understood. The difficulty in using fresh samples of sensory neuronal human fibers limits disclosure of the FD molecular and functional mechanisms. To overcome this limitation was created a transgenic mouse model for the *α*-GalA loss of function [8], [9]. Noteworthy, over the recent years, several ion channels (ICs), receptors, and regulatory proteins involved in pain signaling transduction pathways at the periphery and central nervous system have been investigated by the generation of both KO and transgenic mice [10]–[14]. Specifically, the *α*-GalA deficient mice appear clinically normal but display an evident accumulation of glycosphingolipids between 3 and 6 months of age in several organs, similar to that observed in FD. Interestingly, correction of the enzyme deficit and clearance of the accumulated residues occurred in fibroblasts of the knock out (KO) mouse model [9] which reflect the high level of analogy on the mechanisms in the pathophysiological process of FD patients. Taken together, these data indicate that *α*-GalA KO mice are an excellent system model for the study of FD [3], [9], [15]. Previously, Rodrigues and co-workers [16] demonstrated that this knockout mouse has alterations in sensorimotor function and hypoalgesia. Since experimental evidences of Rodrigues group have shown the importance to investigate this mouse model at the early stages of life, we conducted behavioral, immunohistochemical and molecular studies in animals between 8 and 12 weeks of age. In the present study, we demonstrate for the first time that the *α*-Gal KO male mice present molecular and structural alterations in pain sensation such as heat/cold-hyperalgesia and mechano-hyperalgesia.

## Materials and Methods

### Animals and maintenance

Heterozygous female mice for *α*-*GalA* gene deletion (*α*-*Gal A(+/−*)), JAX strain *B6;129-Gla^tm1Kul^*/J (stock number 003535) and WT male *α*-*GalA(+/+*) were purchased from Charles River Laboratories Italia s.r.l. (Jackson Laboratory; Bar Harbor, ME USA). To obtain both *α*-*Gal A(−/0*) hemizygous male mice and *α*-GalA*(−/−*) homozygous females, we crossed *α*-GalA*(+/−)* female and *α*-*Gal A(−/0*) male mice.The mice were housed in groups of 6 in individually ventilated cages (Tecniplast, Italia) with water and food ad libitum in controlled environmental conditions: lights on from 7.00 am to 7.00 pm, 22±2°C temperature and 65% humidity. We separated the groups of studied animals based on their sex (males and females) to see the differences between sexes and compared them to appropriate age groups of control animals. Behavioral experiments were carried out at the Department of Medical and Clinical Sciences (DIMEC) of the University of Bologna with the approval of the local ethical committee (Veterinary Service of the University of Bologna) and in agreement with the National Animal Welfare Act. All efforts were made to minimize animal suffering and the number of animals used was kept to a minimum by the experimental design. All the procedures followed in this work were in compliance with the European Community Council. Directive of 24 November 1986 (86/609/EEC) and were approved by the Ethical committee of the University of Bologna (prot. N.43.IX/9). Because of the severity is greater in males, we decided to focus our study only on the animal model KO males. The age of animals used for behavioural, molecular and immunohistochemistry experiments was in the range of 8 to 12 weeks.

### Genotyping

Primers used for *α*-GalA were: oIMR_5947_ 5′-AGGTCCACAGCAAAGGATTG-3′, oIMR_5948_ 5′-GCAAGTTGCCCTCTGACTTC-3′, oIMR_7415_ 5′-GCCAGAGGCCACTTGTGTAG-3′. The primers amplified bands of 202 bp for *α*-*GalA*(−/−) females and *α*-*GalA*(−/0) males and 295 bp for *α*-GalA (+/+) females and *α*-GalA(+/0) males. The PCR conditions were: 94°C for 3 minutes, 35 cycles of 94°C for 30 seconds, 64°C for 1 minute and 72°C for 1 minute, final elongation at 72°C for 2 minutes.

### Hot plate

Mice were placed in the experimental room 1 hour before the test. Each mouse was placed into a transparent beaker made of Plexiglas with a height of 60 cm and a diameter of 18 cm to avoid the animals escaping from the plate which temperature was set at 52°C±0.1°C by using a thermo-regulated heated plate (Ugo Basile, Varese, Italy). The time (in seconds) between the placement of the animal and the first response: paw licking/fanning or jumping was measured as latency. A 30 s cut-off was used to prevent tissue damage.

### Cold sensitivity

Sensitivity to a cold stimulus was measured using the acetone test. Mice were placed in the experimental room 1 hour before the test and they were acclimated for 2 hours on a wire mesh (Ugo Basile, Varese, Italy). After acclimation, one drop of acetone was applied to the plantar surface of the hindpaw using a 1 ml syringe. Mice were observed for 2 min after each acetone application. Typical responses from mice at rest but not asleep included lifting of the paw followed by a combination of flicking, licking, biting the paw as well as holding the paw in an elevated position lasting 1 s to one min after cessation of the stimulus and/or movement away from the stimulus area were counted as a positive response. The latency to initiate any discomfort behavior within 2 min after acetone application was used as responses to the cold stimulus. Spontaneous pain behavior that occurred within the first 10 s after acetone application was disregarded as a response to the direct application of the droplet [17]. Three trials were performed on each hindpaw with a 5 min interval between trials.

### Cold plate

For the 0°C cold plate assay, a gel Freez-pack M10 was cooled in a −20°C freezer. Plates were placed on a on a bed of ice and allowed to warm to 0°C as measured by a temperature probe. At these conditions the plates were able to hold constant temperature for approximately 3 h. A clear plexiglass cylinder with a diameter of 7 cm and height of 12 cm was situated on the upper surface of the plate and the mice were placed inside the cylinder. The onset of brisk lifts and/or flicking/licking of the paw were assessed. In these experiments we used the first discomfort sign (i.e, paw licking/fanning or jumping) without distinction between hind- or forepaw. Prior to assay mice were acclimated in an equivalent chamber at room temperature for 2 h. A 60 s cut-off was used to prevent tissue damage.

### Cold plantar assay

The assay of cold sensation was performed as previously described Brenner et al. [18] with following modifications. A 15 mm thick pyrex borosilicate float glass was used to create a working surface. Mice were acclimated on the glass plate in transparent plastic enclosures (Ugo Basile) separated by opaque grey dividers for 2 hours, prior to assay performance.

To make the cold probe, freshly delivered dry ice was crushed into a fine powder using a hammer. To shape the probe, the final end of a 1 ml syringe was cutted and a needle tip was used to make holes on each side of the syringe to prevent gas build up inside the syringe body. The powdered dry ice was packed into the modified syringe and the open end of the syringe was held against a flat surface while pressure was applied to the plunger to compress the dry ice into a flattened, dense pellet 1 cm in diameter.

The onset of brisk hindpaw lifts and/or flicking/licking of the hindpaw was assessed. A 30 s cut-off was used to prevent tissue damage.

### Mechanical hyperalgesia

Paw withdrawal latency to mechanical stimulation was assessed with an automated testing device consisting of a steel rod that was pushed against the plantar surface of the paw with increasing force until the paw was withdrawn (Dynamic Plantar Aesthesiometer, Ugo Basile, Varese, Italy). A linear increase in force to 5 g was applied over 10 s after which the force remained constant at 5 gr for 30 s. To prevent tissue damage the ramp speed was 0.5 g/s. Mice were habituated to the experimental room 1 h before the onset of the test and they were placed in test cages with a metal grid bottom at least 2 h prior to testing to allow accommodation in the novel environment. The paw withdrawal latency and actual force at the time of paw withdrawal reflex were calculated as the mean of 5 consecutive trials.

### Immunohistochemistry

Following behavioral testing male mice of each genotype were deeply anesthetized with Isofluorano and right frontal paws were removed and fixed in 4% paraformaldehyde (Sigma) fixative at 4°C, rinsed in phosphate buffered saline (PBS) (0.01M, pH 7.4) for 4 days, and cryoprotected in 25% sucrose. Paws were mounted in Tissue Tek OCT compound, frozen and cryosectioned at 12 µm transversal to long paw axis and 50 µm parallel with long paw axis (sagittal free floating sections). The immunohistochemistry of floating sections was processed in separated chambers for each genotype. The number of immunohistochemistry experiments was at least 3 per each onset of antibodies and the total number of used animals, was 5. Sections were blocked with 5% bovine serum albumin (BSA) (Sigma) rinsed in PBS (0.01M, pH 7.4) with 0.5% of Triton X-100 (Sigma) for 1 hour at room temperature, followed by incubation overnight with primary antibodies in 1% BSA rinsed in PBS (0.01M, pH 7.4) with 0.05%–0.5% of Triton X-100 at 4°C. After washing they were incubated with secondary antibodies in 1% BSA rinsed in PBS (0.01M, pH 7.4) with 0.05% of Triton X-100 for 2 hours at room temperature. Finally, the sections were incubated with nucleic acid staining and mounted into Fluoromount-G mounting medium (Sigma). Experimental, control and negative control samples, were processed in parallel in different reaction chambers. The fluorescent signal of negative control samples was taken as a threshold for fluorescent signal of experimental and control samples.

Primary antibodies were: rabbit anti-pan-neuronal marker PGP9.5 (1∶1000; Serotec, Raleigh, NC), mouse anti-CollagenIV (1∶800, Chemicon), goat anti-Nav1.8 (1∶50, Santa Cruz), rabbit anti-TRPV1 (1∶50, Immunological Sciences), rabbit anti-TRPM8 (1∶50, Immunological Sciences), rat anti-CD77 (1∶10, Abcam). Secondary antibodies were: Cy2-donkey anti-rabbit, Cy3-donkey anti-mouse, Cy3-donkey anti-rat, Cy3-donkey anti-rabbit, (1∶200, all from Jackson ImmunoResearch) and Cy3-conjugated AffiniPure Fab fragments Donkey anti-rabbit (1∶100, Jackson ImmunoResearch). DAPI (4′,6-diaminobenzidine-2-phenylindole, dilactate; 300 nM, Invitrogen) was used for counterstaining.

### Western blot

Protein extracts were prepared from paw tissues as described previously [19] with the following modifications: from each whole paw was cutted piece of skin, fractioned into small pieces and homogenized with 100 µl of lysis buffer (50 mM TRIS-HCl, pH 7.4, 100 mM NaCl, 1 mM PMSF, 1 mM EDTA, 5 mM Iodacetamide, 1% Triton X-100, 0.5% Sodium dodecysulphate). The extract was sonicated for 10 minutes in 20 seconds intervals every 2 minutes and pelleted for 30 minutes at 12 000 rpm. The supernatant was collected and used to determine the protein content using the Bradford method.

40–80 µg of tissue lysates were separated by 7.5–10% SDS-polyacrylamide gel and transferred to hybond-ECL nitrocellulose membrane (Amersham). After transfer the membrane was blocked by 5% BSA in PBS (0.01M, pH 7.4) with 0.05% Tween 20 (Sigma) (PBST) for 1 hour at room temperature. The membrane was incubated with primary antibodies against specific ionic channels Nav1.8 (1∶200), TRPV1 (1∶500), TRPM8 (1∶250; Immunological Sciences) and TRPM8 (1∶200; Santa Cruz) in 1% BSA in PBST overnight at 4°C. The membrane was rinsed 3 times with PBST, each for 15 minutes and secondary antibodies Horseradish peroxidase-coupled secondary anti-rabbit (1∶1000, Santa Cruz) and anti-goat (1∶5000, Sigma) were employed for incubation in 1% BSA rinsed in PBST for 2 hours at room temperature. After washout of secondary-HRP binding antibody membrane was incubated with chemiluminiscence substrate (Santa Cruz) for 5 minutes; protein bands were visualized on X-ray (Thermo Scientific). The intensity of the protein bands for all observed ICs (TRPV1, Nav1.8 and TRPM8) was quantified and normalized to the intensity of *β*-actin bands using the ImageJ software.

The specificity of each IC signal in this method was assured by membrane incubation with only secondary-HRP binding antibody. The IC positive signal was determined based on presence of specific band of predicted size on the membrane incubated with particular primary antibody and no presence this band on the membrane incubated with only secondary antibody.

### Evaluation of epidermal nerve fiber (ENF) density in mice forepaw glabrous skin

The epidermal nerve fiber (ENF) density identified by PGP 9.5 positive staining was calculated per linear millimeter of epidermis [20]. The images of PGP9.5 and Collagen IV positive immunostaining of forepaw glabrous skin were taken with 40× objective and analyzed using the ImageJ software. There were taken into account only the neuronal fibers within the epidermis, piercing the horizontal band of basal lamina stained for Collagen IV. The total number of counted neuronal fibers was related to the final length of dermal/epidermal border, determined by Collagen IV staining.

### Evaluation of Nav1.8/TRPV1 and PGP9.5 co-localization in neuronal fibers of mice paw glabrous skin

The images were taken at confocal microscope with objective 40× based on the PGP-Cy2 or PGP-Cy3 signal of each channel separately. The signal of PGP was expressed more abundant, because of different penetrance efficiency of primary antibodies Nav1.8, TRPV1 and PGP9.5. The sections selected for stack splitting of separated channels were determined based on Nav1.8-Cy3/TRPV1-Cy2 signal expression. These stacks were proceeded to analyze the co-localization in ImageJ software using plugins: Adaptive Treshold and JaCoP. Each stack was processed with the same level of threshold per particular channel (Cy2 and Cy3 separately). The JaCoP plugin was employed to obtain the Pearson's coefficient to calculate the values automatically. Three to four animals per each genotype (WT and KO) were used. Paw sections of 50 µm were stained via regular procedure of double immunohistochemistry and 8 sections were selected for analysis with the couple of antibodies anti-rabbit PGP with anti-goat Nav1.8. For immunohistochemistry of anti-rabbit PGP with anti-rabbit TRPV1 were employed Fab-Cy3 conjugated fragments coupled with PGP 9.5 antibody. For consecutive staining with anti-rabbit TRPV1 were used Cy2 conjugated goat and donkey anti-rabbit secondary antibodies. The co-localization points are visualized as 8bits white channel using the ImageJ plugin.

### Quantification of TRPV1, PGP9.5 and Nav1.7 fluorescence expression

The fluorescent intensity of TRPV1, PGP9.5 and Nav1.7 expression was calculated as previously described by Burgess et al. [21]. The fluorescent intensity of TRPV1 and PGP9.5 expression in WT and KO was determined in single fibers entering the epidermal part of mice frontal paw glabrous skin, meanwhile the fluorescent intensity of Nav1.7 was measured in different areas of dermis and the value of fluorescence was related to the area of its expression. The fluorescent intensity of TRPV1 was relativized to the total number of fibers per mm per each genotype.

### Data acquisition

Fluorescent images were captured on a Nikon D-Eclipse C1 inverted laser scanning confocal microscope. The images were taken as stacks of 20–30 µm every 0.5 µm, separately for each channel (for co-localization evaluation) or as single confocal sections (for ICs expression demonstration) all taken separately for each channel. EZ-C1 3.90 FreeViewer, Image J (NIH, http://rsb.info.nih.gov/ij/) and Adobe Photoshop software were used for image capture and analysis.

### Statistical analysis

Quantity One program and ImageJ (gel analyser plugin) were used for quantification of western blots and GraphPad Prism software was used to perform an unpaired Student's *t*-test for statistical significance. Values shown represent mean±SEM. A p-value of <0.05 (*), p<0.01 (**) and p<0.001 (***) were chosen as indicating significance. Quantification of behavioral experiments was based on comparing of two groups WT and KO males. For this analysis was employed GraphPad Prism software, using unpaired Student's *t*-test. A p-value of <0.05 (*), p<0.01 (**) and p<0.001 (***) were chosen as indicating significance. A test of Homogeneity of Variances (Levene's test) was run to check the parametrical distribution of the data. In case of no-parametrical distribution, data were analyzed using a Mann Whitney test. A p-value of <0.05 (*), p<0.01 (**) and p<0.001 (***) were chosen as indicating significance.

## Results

### Effect of genotype on weight

**Figure 1:**
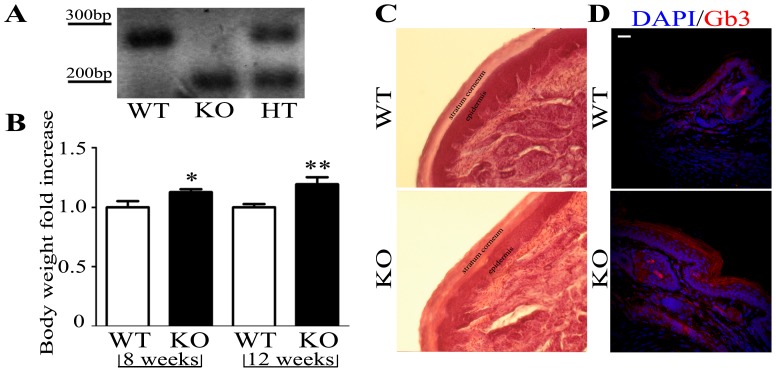
The genotypic, anatomical and immunohistochemistry characterization of *α*-GalA KO mice. The specific primers amplified bands of 295 bp for *α*-GalA(+/+) WT and 202 bp for *α*-GalA(−/−) and *α*-GalA(−/0) KO. In case of founders females the heterozygosity was confirmed by PCR amplification of both WT and KO bands (A). The body weight of Fabry males was significantly increased just after 8 weeks (n = 15 for WT, n = 20 for KO; *p = 0.0111*). This trend was maintained and even increased after 12 week of age (n = 10 for WT, n = 5 for KO; *p = 0.0023*). Data are presented as fold of body weight increase in KO males related to the mean of WT males (B). Hematoxylin-eosin staining of 12 µm frozen frontal paws sections (C). Immunohistochemistry of frontal paw sections clearly shows the accumulation of globotriaosylceramide (Gb3; red) in the epidermis and stratum corneum of KO in comparison to WT mice (D). Scale bar represents 100 µm. Graphical data are expressed as mean±SEM.

We first determined the genotype of our colony. Tails DNA from offspring mice were extracted and genotyped by PCR amplification, using three oligonucleotide primers: the WT locus amplified a band of 295 bp, the targeted locus amplified a band of 202 bp, whereas the heterozygous females were distinguished by the presence of both amplified bands (Figure 1A).

Further, we examined the body weight of the *α*-GalA(−/−) and WT animals at 8 and 12 weeks of age. Interestingly, male FD mice *α*-GalA(−/0) showed a significant increase in body weight compared to their control groups after 8 weeks (Figure 1B *p_8weeks_ = 0.0111*; n = 15 animals for WT, n = 20 animals for KO). This increase was even more significant after 12 weeks (*p_12weeks_ = 0.0111*, n = 10 animals for WT, n = 5 animals for KO) suggesting that the accumulation of Gb3 in diverse organs of FD male (*α*-GalA(−/0)) body leads to an increment in weight. In contrast, FD female mice *α*-GalA(−/−) did not show significant differences (Figure S1 *p_8weeks_ = 0.3771*; n = 13 animals for WT, n = 7 animals for KO and *p_12weeks_ = 0.1510*; n = 9 animals for WT, n = 2 animals for KO). Since the severity is greater in males, we decided to focus our study only on the animal model KO males. To deeply characterized the morphology of the *α*-GalA(−/−) skin mice model, hematoxylin and eosin-staining of frontal paws sections was employed for anatomical detection of *α*-GalA(*−/0*) and *α*-GalA*(+/+)* male mice (Figure 1C). Moreover, figure 1D shows the accumulation of Gb3 expression in frontal paw epidermis and stratum corneum of KO male mice compared to control.

### Mechanical sensitivity of *α*-GalA deficient mice

**Figure 2:**
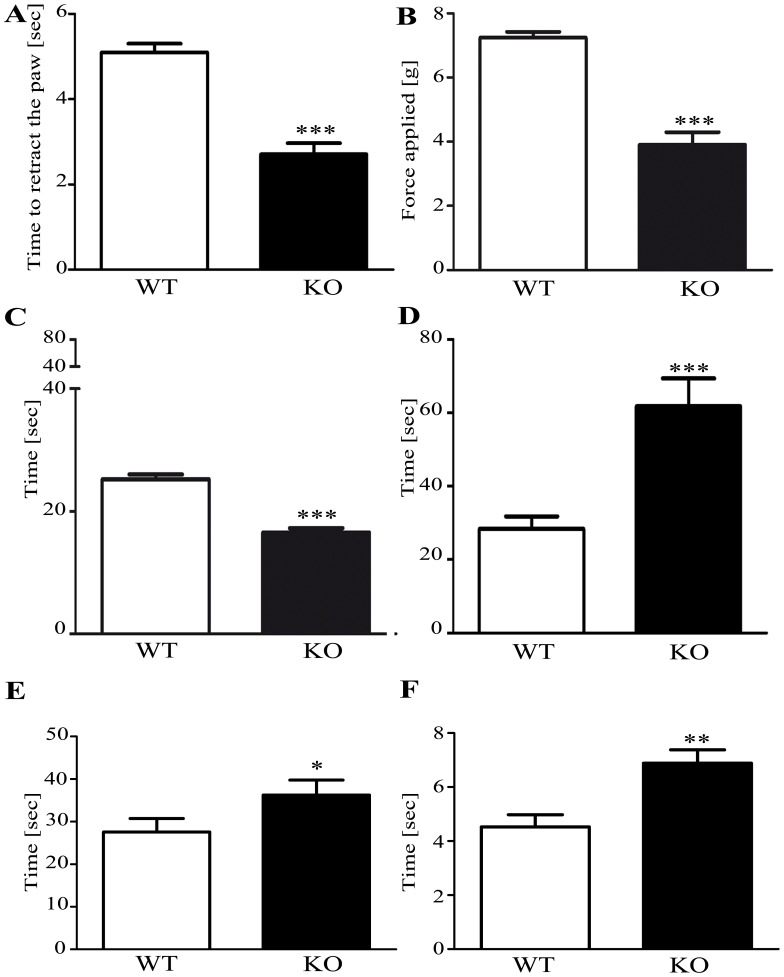
Mechanical and thermal sensitivity of *α*-GalA KO males. Basal sensitivity towards mechanical stimulation (latency time (A); applied force (B)). Comparison of the basal sensitivity of male *α*-GalA KO (n = 16) and relative WT (n = 28) in response to mechanical stimulation, *p<0.0001*. Basal sensitivity to hot and cold temperature stimuli in male *α*-GalA KO (n = 34) and relative WT (n = 32), *p<0.0001* as measured using the hot plate (C) and the acetone test (D) revealed the significant insensitivity of *α*-GalA KO (n = 19) males in comparison to their relative WT (n = 13), *p = 0.0008*. Basal insensitivity to noxious temperature of cold stimulus in male *α*-GalA KO (n = 7) and relative WT (n = 8), *p = 0.0466* as measured via cold plate assay (E). The data from plantar cold sensitivity assay confirmed the observed insensitivity of KO males (n = 10) to cold stimuli when compared to WT males (n = 10), *p = 0.0028* (F). Data are expressed as mean±SEM.

To determine whether the loss of activity of *α*-galactosidase A (*α*-GalA) affects mechanical sensitivity, an automated von Frey test was performed on *α*-GalA*(−/0)* males and control mice. Two parameters were analysed: latency time to paw withdrawal and the force of the stimulus. There was a significant difference in withdrawal threshold (reaction time and force) to a mechanical stimulus between KO and control (WT) mice (Figure 2A and B *p<0.0001*, n = 28 animals for WT, n = 16 animals for KO). This decrease in withdrawal threshold to a mechanical stimulus could be due to a hyperalgesic state induced by the loss of activity of *α*-GalA.

### Thermal sensitivity of *α*-GalA mice

To study the sensory function of FD regarding to acute nociceptive response evoked by hot and cold painful temperature stimuli, a hot-plate, an acetone test, cold plate and a noxious plantar cold test were respectively performed. Since Rodrigues et al. 2009 obtained no response from the KO Fabry mice up to approximately 50°C, so far we decided for the stimulus at 50–52°C using the standard hot plate test [16]. We assessed the first discomfort sign (i.e, paw licking/fanning or jumping) without distinction between hind or forepaw as a signature of pain.

We found a statistical significant reduction in latency time between male KO and male WT groups demonstrating more sensitivity to noxious hot thermal stimulation than control (Figure 2C *p<0.0001*, n = 32 animals for WT, n = 34 animals for KO). The hot plate test was also carried out in groups of KO and WT males with 8 months of age resulting in a decreased level of significance (Figure S3), similar to previously described by Rodrigues et al., 2009.

To determine whether loss of activity of *α*-GalA*(−/0)* influences sensitivity also to a cold stimulus, we employed three different cold sensation assays. Our data from acetone test performed on hindpaw show that males *α*-GalA*(−/0)* are less sensible to cold stimulation since we found a significant increase in latency time after an acetone applications compare to male control mice (Figure 2D *p = 0.0008*, n = 13 animals for WT, n = 19 animals for KO). The data from the cold plate experiments (Figure 2E *p = 0.0466*, n = 8 animals for WT, n = 7 animals for KO), in which we assessed the first discomfort sign such as forepaw lifts and/or flicking/licking), confirmed the decreased sensitivity of *α*-GalA*(−/0)* male to cold stimulus caused by 0°C when compared with *α*-GalA*(+/+)* males. In addition, in order to discriminate between innocuous and noxious cold, we performed a more effective noxious test of plantar cold sensitivity. Interestingly, the *α*-GalA*(−/0)* males showed again a statistically low cold thermal sensitivity compared to the control mice (Figure 2F *p* = *0.0028*, n = 10 animals for WT, n = 10 animals for KO).

### Nociceptive circuitry in *α*-*GalA(−/0) mice*

**Figure 3:**
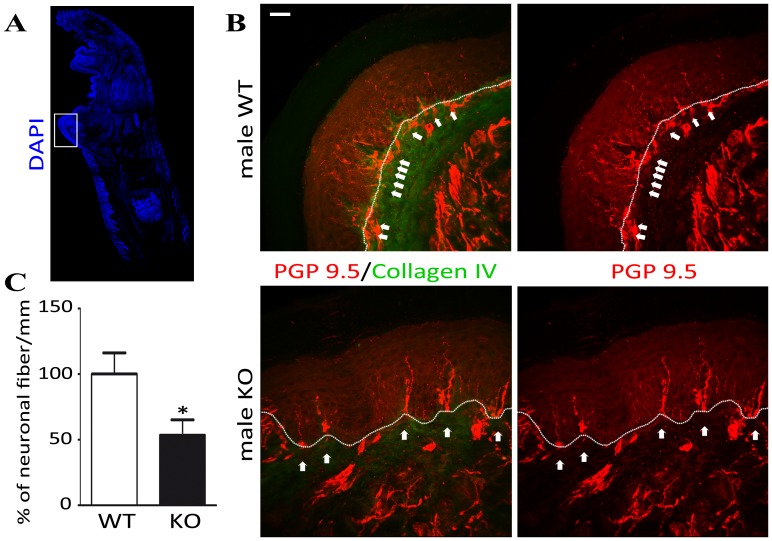
Detection of ENFs in *α*-GalA KO males frontal paws. The DAPI immunostaining of 50 µm floating sagittal mice frontal paw section with marked region of interest (A). The immunohistochemistry of *α*-GalA KO males (n = 3) revealed the scattered expression of PGP9.5 (red) - specific marker of neuronal terminations in the epidermis of frontal paw skin in comparison to their WT controls (n = 3). The dermis and epidermis border was distinguished by staining for Collagen IV (green) and visually determined by dotted lines. Paw epidermal PGP9.5 positive fibers showed morphological abnormality such as fragmentation in *α*-GalA KO males, whereas the epidermal fibers showed a more regular morphology in WT males (white arrows) (B). Scale bar represents 100 µm. Numerical analysis of neuronal fibers terminations showed significant decrease (about 50%; *p = 0.0161*) in *α*-GalA KO males in comparison to WT (C).

The epidermis of mammalian non-hairy (glabrous) skin is densely innervated by nociceptor endings that carry harmful chemical, mechanical and temperature stimuli [22], [23]. The immunohistochemistry of floating longitudinal sections of frontal mice paw revealed that neuronal fibers in dermis of glabrous skin were present in lower abundance in *α*-GalA*(−/0)* individuals compared to control mice. Notably, density of the ENFs was markedly decreased in KO mice compared to WT (37% less PGP 9.5 positive fibers in KO (5.11 fibers/mm) in comparison to WT (9.58 fibers/mm) (Figure 3 *p = 0.0161*, n = 3 animals for WT, n = 3 animals for KO). Moreover, in the case of *α*-GalA*(−/0)* mice, we found morphological abnormalities such as nerve fibers swelling and fragmentations (Figure 3, white arrows). Interestingly, the reduction of PGP 9.5 immunostaining was not limited to the epidermis but also involved the sub-epidermis plexus.

### Protein expression of ionic channels specific for neuropathic pain in mice frontal paw

After the behavioral studies the same mice were used for histological staining of protein expression of the transient receptor potential cation channel subfamily V member 1 (TRPV1), voltage-gated sodium channel (Nav1.8) and transient receptor potential cation channel subfamily M member 8 (TRPM8) cationic channels.

**Figure 4:**
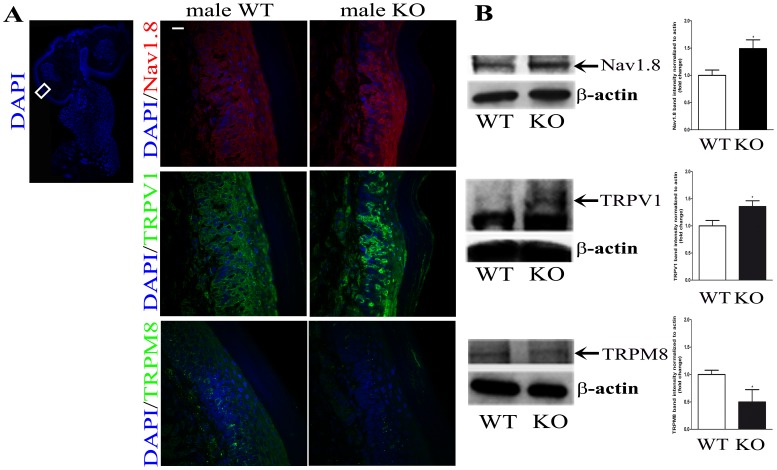
The expression of pain receptors in *α*-GalA KO males frontal paws. A) Immunohistochemistry of 12 µm frozen coronal sections of α-GalA KO males (n = 3) showed different expression at the protein level of the specific pain (Nav1.8), heat (TRPV1) and cold (TRPM8, Immunological Science) receptors in comparison to their WT controls (n = 3). B) The western blot analysis of Nav1.8 expression (∼220 kDa) in the whole protein extract from skin of frontal paws confirmed 1.5 fold increased protein expression of Nav1.8 in case of α-GalA KO males (n = 5) when compared to WT males (n = 5), p = 0.0246. The western blot analysis of TRPV1 expression (∼92 kDa) revealed 1.4 fold increased protein expression in case of α-GalA KO males (n = 3) when compared to WT (n = 3), p = 0.0344. The analysis of TRPM8 (Santa Cruz) expression (∼127 kDa) in the whole protein extract from skin of frontal paws showed the almost half expression decrease in protein expression of TRPM8 in case of α-GalA KO (n = 3) males when compared to WT (n = 3), p = 0.0518. Original images of presented blots are part of supplementary material (Figure S4). Scale bar represents 100 µm.

About 50% of C-fiber and 10% of A*δ* fibers in naive rats express Nav1.8 channels [24]. This percentage is increased in conditions of pain [25], [26]. More Nav1.8 channels are expressed on C-fibers and A*δ* fibers in rat digital nerves after neuropathic injury [27]. In our immunological studies we have observed increased expression of Nav1.8 in epidermis of frontal paw glabrous skin in case of *α*-GalA*(−/0)* mice in comparison with control (Figure 4A, n = 3 animals for WT, n = 3 animals for KO). The western blot analysis of Nav1.8 expression in the whole protein extract from skin of frontal paws confirmed that the ratio of Nav1.8 protein to *β*-actin was higher in KO males respectively to their control. Specifically, the higher protein expression of Nav1.8 in case of *α*-Gal A KO males is about 1.5 fold increase compared to WT (Figure 4B *p = 0.0246*, n = 5 animals for WT, n = 5 animals for KO). Interestingly, the expression of the voltage-gated sodium channel Nav1.7 which plays important roles in inflammatory and neuropathic pain remained unaffected in control and FD mice (Figure S2, n = 2 animals for WT, n = 2 animals for KO, *p = 0.1495*).

TRPV1 is a non-selective cation channel which responds to a variety of different stimuli. One of these stimuli is the activation by temperature greater than 43°C. Our behavioral experiments using the Hot Plate technique showed significantly increased sensitivity of males *α*-GalA(−/0) mice in comparison to controls. We therefore carried out an immunohistochemistry analysis for expression of TRPV1 in glabrous skin of frontal paw of KO and WT males. As figure 4A shows, we observed an important increase of TRPV1 expression *α*-GalA*(−/0)* males in comparison to WT. This finding was independently confirmed by quantitative western blot analysis. Again, protein expression analysis revealed a 1.4-fold increase in TRPV1 signal in the total protein extraction from mice hind paw from KO compared to the control (WT) (Figure 4B *p = 0.0344*, n = 5 animals for WT, n = 5 animals for KO).

The cold sensitivity test by acetone clearly showed an increased insensitivity of *α*-GalA(−/0) mice to cold. To support this observation we employed immunohistochemistry for detection of TRPM8 protein level. As figure 4A shows, there is a decrease in the expression of TRPM8 protein in transversal sections of glabrous skin of frontal paw of *α*-GalA(−/0) mice in comparison to WT. The quantitative western blot analysis revealed a significant decrease in TRPM8 expression in KO compared to the control males (about half expression decrease) (Figure 4B *p = 0.05*, n = 5 animals for WT, n = 5 animals for KO).

### Co-localization of Nav1.8 and TRPV1 with the epidermal nerve fibers

**Figure 5:**
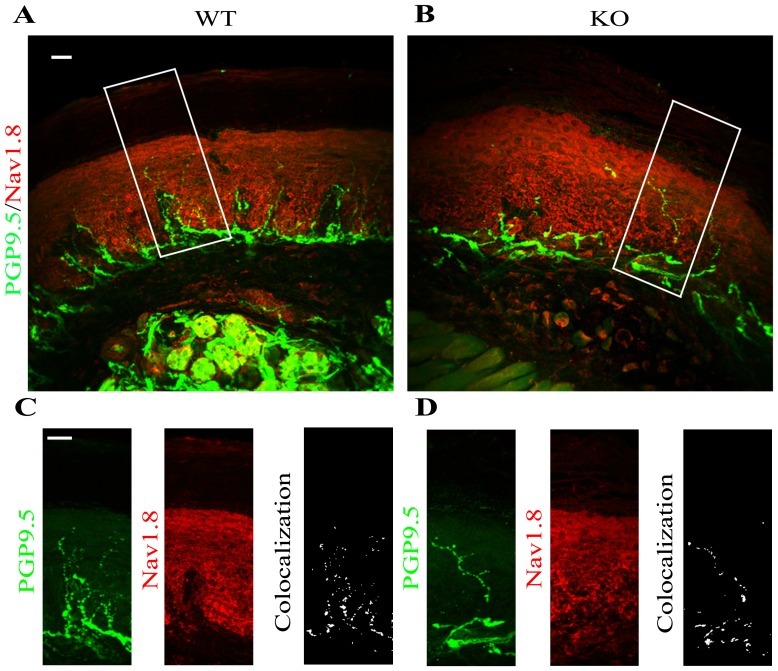
The expression of Nav1.8 in epidermal neuronal fibers of *α*-GalA KO males frontal paws. The evaluation of co-localization of Nav1.8 and PGP 9.5 expression in 50 µm floating sagittal sections of WT (A) and *α*-GalA KO males (B) (n = 3) revealed similar values of Pearson's coefficient in both cases (*P_WT_ = 0.16*, *P_KO_ = 0.12*). The neuropathic pain receptor Nav1.8 is expressed in neuronal fibers of WT (C left panel) males marked by specific antibody PGP 9.5 with the same intensity as it is expressed in *α*-GalA KO (D left panel, *p = 0.1911*). Panels C, D were enhanced with 1.125 zoom respectively to square area in figures A, B. Scale bars represent 100 µm.

Cumulative evidences have demonstrated that peripheral inflammation increases the expression of Nav1.8 in dorsal root ganglion (DRG) neurons, suggesting that they participate in the induction and maintenance of chronic inflammatory pain [25], [28]. To determine whether the increase in pain perception in *α*-Gal A(−/0) mice was due to an increased expression of ionic channels involved in sensory pain transduction, we next addressed the co-localization between epidermal endings of PGP 9.5 positive neuronal fibers in glabrous skin of frontal paw of KO and WT and Nav1.8 ion channel. Average of 8 values of Pearson's coefficients was calculated per each genotype (Fig. 5, WT A–C or KO B–D *p = 0.1911*, n = 3 animals for WT, n = 3 animals for KO). *P_average_* of WT is *P_WT_ = 0.16*, *P_average_* of KO is *P_KO_ = 0.12*. These values correspond to the range of Pearson for small correlation, where P is between 0.1 and 0.3. Due to the values of P_WT_ = 0.16 and P_KO_ = 0.12 we can conclude that the co-localization of Nav1.8 and PGP in neural fibers of mice paw glabrous skin was similar for WT and KO mice and no significant differences were observed by using the Pearson correlation coefficient.

**Figure 6:**
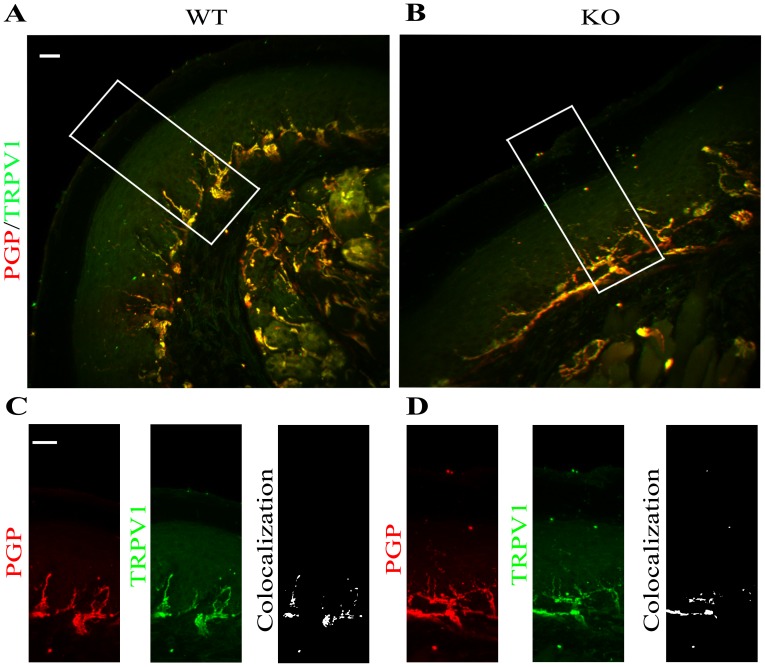
The expression of TRPV1 in epidermal neuronal fibers of *α*-GalA KO males frontal paws. The evaluation of co-localization of TRPV1 and PGP 9.5 expression in 50 µm floating sagittal sections of WT (A) and *α*-GalA KO (B) males (n = 4) revealed similar values of Pearson's coefficient in both *α*-GalA WT males (*P_WT_ = 0.35*) and KO (*P_KO_ = 0.29*), (*p = 0.1132*). The TRPV1 receptor is expressed in neuronal fibers of WT (C left panel) males marked by specific antibody PGP 9.5 with the high intensity as it is expressed in *α*-GalA KO (D left panel). The co-localization of both markers (C, D right panels) revealed similar results. Panels C, D were enhanced with 1.125 zoom respectively to square area in panels A, B. Scale bars represent 100 µm.

Next, we examined whether the activation of high temperature perception pathway revealed by the behavioral experiment was due to an over expression of TRPV1 receptor specifically in the epidermal nerve fibers. To this end, we performed co-localization analysis between epidermal endings of PGP 9.5 positive neuronal fibers glabrous skin of frontal paw of KO and WT and TRPV1 receptors (Figure 6 *p = 0.1132*, n = 3 animals for WT, n = 3 animals for KO). The Pearson correlation coefficients expressed as a P value for *P_WT_ = 0.35* and *P_KO_ = 0.29* suggest that the co-localization of TRPV1 and PGP 9.5 in neural fibers of *α*-GalA(−/0) mice paw glabrous skin was similar in both investigated groups of animals. On the contrary, TRPM8 was omitted since the strong decrease of receptor detection in the *α*-GalA KO mouse model.

## Discussion

*α*-GalA deficiency causes a pleiotropic phenotype which in humans is known as Fabry Disease. In the present work, our main goal was to determine to what extent the alterations of phenotypic traits in mice mutant for this enzyme recapitulate the alterations found in the human disease. To this end, we have used the *α*-GalA KO mice to characterize different phenotypic, morphological and molecular parameters altered in FD. Due to the X-chromosomal inheritance of FD, heterozygous female carriers can be asymptomatic or clinically affected, usually with a late onset and mild form of the disease [3]. In this context, the most affected males have little, if any, *α*-GalA activity and replicate completely the clinical manifestation of the disease including angiokeratoma, hypohydrosism, renal failure and pain crises [1]. Nevertheless, it is important to highlight that recently published articles, reflect the shift in this classical view of the expression of FD in females, showing a high number of heterozygous females manifesting clinical signs ranging from mild to very severe frameworks, similar to those found in affected males. From this point of view, in women the age of onset and evolution are generally delayed [29]. This extreme variability depends on the different configurations of X-chromosome inactivation which is tissue-specific and the organ damage in women depends on the degree of mosaicism between healthy and altered cells. This leads to a residual variability in *α*-GalA enzyme activity, in serum or leukocytes [30], . The hemizygous males *α*-Gal*A(−/0)* and homozygous females *α*-GalA*(−/−)* display significant accumulation of Gb3 in their kidneys, hearts and livers in comparison to WT mice, which express the detectable level of Gb3 accumulation only in kidneys. Even though the accumulation of Gb3 in FD model is detectable in different organs, this phenomenon is not observed in the brains. The rate of Gb3 accumulation reaches important significance between 4 and 16 weeks, with persistency till 52 weeks of age [32]. Interestingly, Rodrigues et al. (2009) found that FD show significantly increased body weight after 24 and 48 weeks in comparison to control WT mice. The same authors claimed about neuronal alterations in FD mice that are difficult to detect at reported ages. Moreover, when 8 week-old *α*-GalA*(−/0)* males were treated by a potent inhibitor of glucosylceramide synthase for 4 weeks, significant changes in the body weight loss were observed. This suggests that the used inhibitor caused decreasing of Gb3 levels down the levels of non-treated KO males [32]. Therefore, in our studies we have used male mice of 8–12 weeks age and our results show significant body weight increase already after 8 weeks. This increment was even more significant after 12 weeks, what leads to conclusion that the accumulation of Gb3 in diverse organs of FD mice body leads to gain of weight. FD *α*-GalA*(−/−)* female mice did not show such a significant difference in any of the observed time period (Figure S1). Taken together, based on previously described observations [16], [29], [32] and our results on *α*-GalA*(−/−)* females, we decided to focus our detailed analysis exclusively on *α*-GalA*(−/0)* males.

Mechanical and thermal sensitivity of *α*-GalA KO males were analyzed by Dynamic Plantar Aesthesiometer (Electronic von Frey) and Hot-Plate tests. Both tested parameters, force and time in electronic von Frey analysis, revealed very significant differences between the two groups. Our results clearly show the higher mechanical sensitivity of *α*-GalA*(−/0)* males manifested by the shorter time of latency to hind paw withdrawal in comparison to control males. In parallel, we observed a compelling increase in touch sensitivity, revealed by a decrease in the grams (g) of applied pressure required to elicit response.

The hot plate assay is one of the most commonly used tests for determining the antinociceptive efficacy of experimental drugs in rodents [28], [33], [34]. Different members of TRP channel family are activated in different ranges of heat temperature. Since Caterina et al. [35] first time cloned and characterize TRPV1 as an heat-gated ion channel with ≥42°C activation threshold, two groups provided the targeted deletion of *trpv1* gene in order to produce knockout TRPV1−/− mice [36], [37]. The responses to noxious heat stimuli in TRPV1−/− mice become apparent only at temperatures over 50°C. At the threshold of our Hot Plate assay (52°C) could be possibly also activated another TRP channel TRPV2 (VRL1) [38] but we focused our interest on the TRPV1 expression. It should be noticed that, usually, hot-plate analgesia measures the latency for the rodent to lift and/or lick a hindpaw from the floor of the apparatus, which is heated to a temperature of 52–55°C [39]. Our conclusions assessed from the acute pain sensitivity to a thermal stimulus revealed the increased sensitivity of KO males to heat temperature stimulus in comparison to WT. This observation support the observation obtained from human behavioral testing of Fabry patients, where the hyperalgesia was observed.

Recently, dysfunctions of different ICs widely distributed in human sensory neurons Aβ and C-fibers have been proposed to be involved in pain transmission and sensation in SFNs [40], [41]. Specifically, the sodium channels named Nav1.7, Nav1.8 and Nav1.9 widely distributed in human sensory neurons of A delta (Aδ) and C-fibers has been shown to be a keynote in generating and maintaining the action potential in damage-sensing sensory neurons and have been linked to pain pathways [42]–[44]. In this context, Minnet et al., 2012, 2014 [43], [45] have demonstrated that phenotypically identical pain syndromes are induced through different molecular mechanisms in distinct sets on sensory and sympathetic neurons. Notably, there are now evidences for a key role of Nav1.8 in controlling the excitability A*β*-fiber excitability and for a potential contribution to the development of mechanical allodynia under persistent inflammation [46]. In addition, studies of families with autosomal dominant erythermalgia show that they bear mutations in the gene codifying for the voltage-gated sodium channel Nav1.7, which is also involved in cases with idiopathic SFN [47], [48].

This finding provided a mechanistic explanation for the role of the voltage-gated sodium channels in pain signaling/transmission and suggests that Nav1.7 and Nav1.8 channels could be relevant to acquired as well as to inherited channelopathies [49], [50]. Leo and co-workers [51] showed that Nav1.8 and Nav1.9 play important roles in thermal allodynia. The role of Nav1.8 in mechanical pain was demonstrated by reducing of Nav1.8 expression by blocking mRNA transcription using antisense oligonucleotides [52]. This technique reduced the mechanical allodynia and hyperalgesia [53]. In this context, our results demonstrate the elevated expression of Nav1.8 in the epidermis of glabrous skin of *α*-GalA*(−/0)* males frontal paws in comparison to expression of Nav1.8 in control WT group. Interestingly, the expression of the neuronal marker PGP9.5 specific for neuronal terminations in the skin showed a significant decreased and scattered pattern of neuronal terminations in *α*-GalA*(−/0)* males. This parallels the observations of a decrease in neuronal terminations marked by PGP9.5 in skin biopsies of patients with small fiber neuropathies [6], [54]. It should be noted, as we showed in figure 3 that the expression of epidermal neural fibers in case of Fabry KO mice is diminished about 47%. The co-localization experiments pointed out that Nav1.8 and PGP9.5 co-localize at the same level in WT and KO mice. It has to be highlighted that the increased expression of Nav1.8 seems to be more abundant in the dermal part of skin than in neuronal fibers. This statement justly leans on already published results of presence of sodium channels in non-excitable cells such as fibroblasts and keratinocytes [44], [55]–[58].

Moreover, the increased expression of transient receptor potential channel (TRP) channels in fibroblast and Na channels in sensory fibers has been shown to participate in nerve fiber transmission of pain [59]–[63]. Several experimental evidences carried in KO mice have demonstrated the involvement of TRPV1 channel in pain sensation [64], [65]. Based on the fact that capsaicin activates TRPV1 receptor is expressed exclusively in C-fibers, FD patients have been used to evaluate the loss of small-fiber function by reduction of allodynia and skin blood flow after topical capsaicin administration [66]. The TRPV1 receptor has been linked in transduction of various stimuli, such as thermosensation, leading to pain sensation [67]. As previously described, our behavioral data show that the *α*-GalA(−/0) males manifested a higher sensibility to temperature of 52°C in comparison to control males by quicker responses such as licking/fanning of frontal paws or jumping. This increase in thermal sensitivity correlates with the increased expression of one of the thermo-sensitive ionic channels, specifically TRPV1.

The immunostaining and protein quantification using the TRPV1 antibody demonstrated increased expression of receptor in the epidermis of glabrous skin of *α*-GalA(−/0) male compared to *α*-GalA(+/+) males. It is important to quote that the 47% decrease of PGP9.5 positive fibers in case of KO mice, has a strong impact on the evaluation, as it was in case of Nav1.8 and PGP9.5 co-localization.

In this context, the TRPV1 receptor signal strongly co-localizes with PGP9.5 expression at the similar level in WT and KO mice. Nevertheless, we hypothesize that the diminished expression of PGP9.5 as a result of swelling and fibers fragmentations at the epidermal and sub-epidermis plexus parallels the increment of TRPV1 receptor in the remained fibers. Therefore we confirmed this hypothesis by the evaluation of TRPV1 intensity expression proportioned to the total number of PGP9.5 positive fibers in WT and KO males (*p* = 0.0356).

The transient receptor potential melastatin 8 channel (TRPM8) plays a clear physiological role in the detection of low temperature ambient (threshold around 25°C) and cooling compounds such as menthol [68]. Herein, the cold sensitivity was confirmed in the *α*-GalA*(−/0)* by three independent action cold tests [68], [69]. The acetone test was used to measure mild sensitivity to cold (approximately 10°C), and results clearly showed an increased latency time after acetone application in case of *α*-GalA*(−/0)* males was significantly longer than in the case of *α*-GalA*(+/+)* males. The cold plate test revealed the significantly decreased sensitivity to 0°C temperature of *α*-GalA*(−/0)* male forepaws in comparison to their control group. Finally, because both above mentioned cold sensitivity tests are limited (both tests reveal the magnitude of response, but do not assess the precise response to the particular temperature [18]) the cold plantar assay was processed. The result of this precise cold assay confirmed the decreased sensitivity of *α*-GalA*(−/0)* males to cold stimulus in comparison to *α*-GalA*(+/+)* males responses. Anyway, we observed that *α*-GalA(−/0) male spent more time by licking the forepaws or shaking/licking the dry ice exposed hindpaw. It could be speculated that this discomfort behavior could indicate a longer perception of pain in KO male compare to WT males. To support the observation of decreased cold sensitivity in *α*-GalA(−/0) males, we employed immunohistochemistry for detection of one of the cold-channel protein expression. The decrease TRPM8 protein level in the epidermis of frontal paws correlates with the decreased sensibility to cold stimuli in FD males. Taken together, we show the utility of *α*-GalA*(−/0)* transgenic males as a suitable model for behavioral testing of pain and sensitivity of FD in rodents. Consequently, our data may contribute to shed light on neural and extra neural mechanisms underlying pain and abnormal sensory transduction characterizing FD.

## Supporting Information

Figure S1 The body weight analysis of *α*-GalA females. No difference was observed in body weight of Fabry *α*-Gal (−/−) and control *α*-Gal (+/+) females after 8 weeks (n = 13 for WT, n = 7 for KO; *p = 0.3771*) and 12 weeks (n = 9 for WT, n = 2 for KO; *p = 0.1510*). Graphical data are expressed as mean±SEM.
(TIF)

Figure S2 The detection and evaluation of Nav1.7 expression in *α*-GalA KO males frontal paws. Immunohistochemistry of 12 µm frozen coronal sections of *α*-GalA KO males (n = 3) showed similar expression at the protein level of the Nav1.7 pain receptor (green) in comparison to their WT controls (n = 3) (A). Scale bar represents 100 µm. Graphical interpretation of Nav1.7 fluorescence quantification (*p = 0.1459*) (B). Graphical data are expressed as mean±SEM.
(TIF)

Figure S3 The hot-plate results after 8 months age of *α*-GalA KO males. Basal sensitivity to noxious temperature stimuli in males of 8 months *α*-GalA KO (n = 34) and relative WT (n = 23), *p<0.0001* as measured with the hot plate (at 52°C). Data are expressed as mean±SEM.
(TIF)

Figure S4 Original WB images. To detect the Nav1.8 (∼220 kDa) protein expression were used 80 µg of tissue lysates separated by 7.5% SDS-polyacrylamide gel (A), for TRPV1 (∼92 kDa) protein expression 40 µg of tissue lysates were separated by 10% SDS-polyacrylamide gel (B), and for TRPM8 (∼127 kDa) protein expression were used 40 µg of tissue lysates separated by 10% SDS-polyacrylamide gel (C). After transfer the membranes were blocked for 1 hour at room temperature and incubated with primary antibodies against specific ionic channels Nav1.8 (1∶200, Santa Cruz), TRPV1 (1∶500, Immunological Sciences) and TRPM8 (1∶200; Santa Cruz) and β-actin (1∶200, Sigma) in 1% BSA in PBST overnight at 4°C. The membranes were rinsed 3 times with PBST, each for 15 minutes and secondary antibodies Horseradish peroxidase-coupled secondary anti-rabbit (1∶1000, Santa Cruz) for TRPV1, TRPM8 and β-actin and secondary anti-goat (1∶5000, Sigma) for Nav1.8 were employed for incubation in 1% BSA rinsed in PBST for 2 hours at room temperature. After washout of secondary-HRP binding antibody membrane was incubated with chemiluminescence substrate (Santa Cruz) for 5 minutes; protein bands were visualized on X-ray (Thermo Scientific).
(TIF)
