# Supplementary figures and images for: Pain Related Channels Are Differentially Expressed in Neuronal and Non-Neuronal Cells of Glabrous Skin of Fabry Knockout Male Mice
Source: PLoS One. 2014 Oct 22;9(10):e108641. doi: 10.1371/journal.pone.0108641 (PMC4206276; doi:10.1371/journal.pone.0108641)

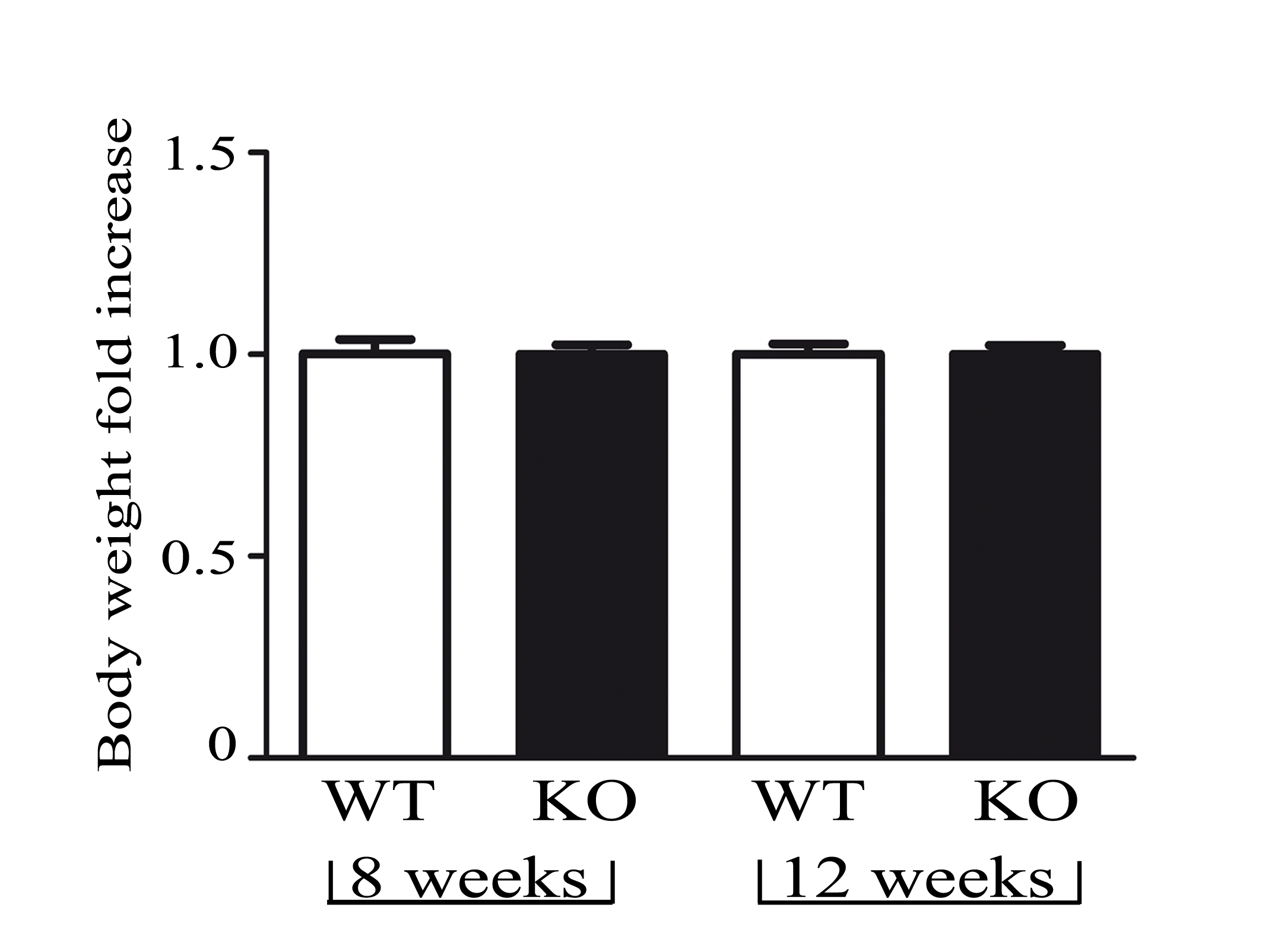

Supplement: Figure S1 — The body weight analysis of α-GalA females. No difference was observed in body weight of Fabry α-Gal (−/−) and control α-Gal (+/+) females after 8 weeks (n = 13 for WT, n = 7 for KO; p = 0.3771) and 12 weeks (n = 9 for WT, n = 2 for KO; p = 0.1510). Graphical data are expressed as mean±SEM. (TIF) [file pone.0108641.s001.tif]

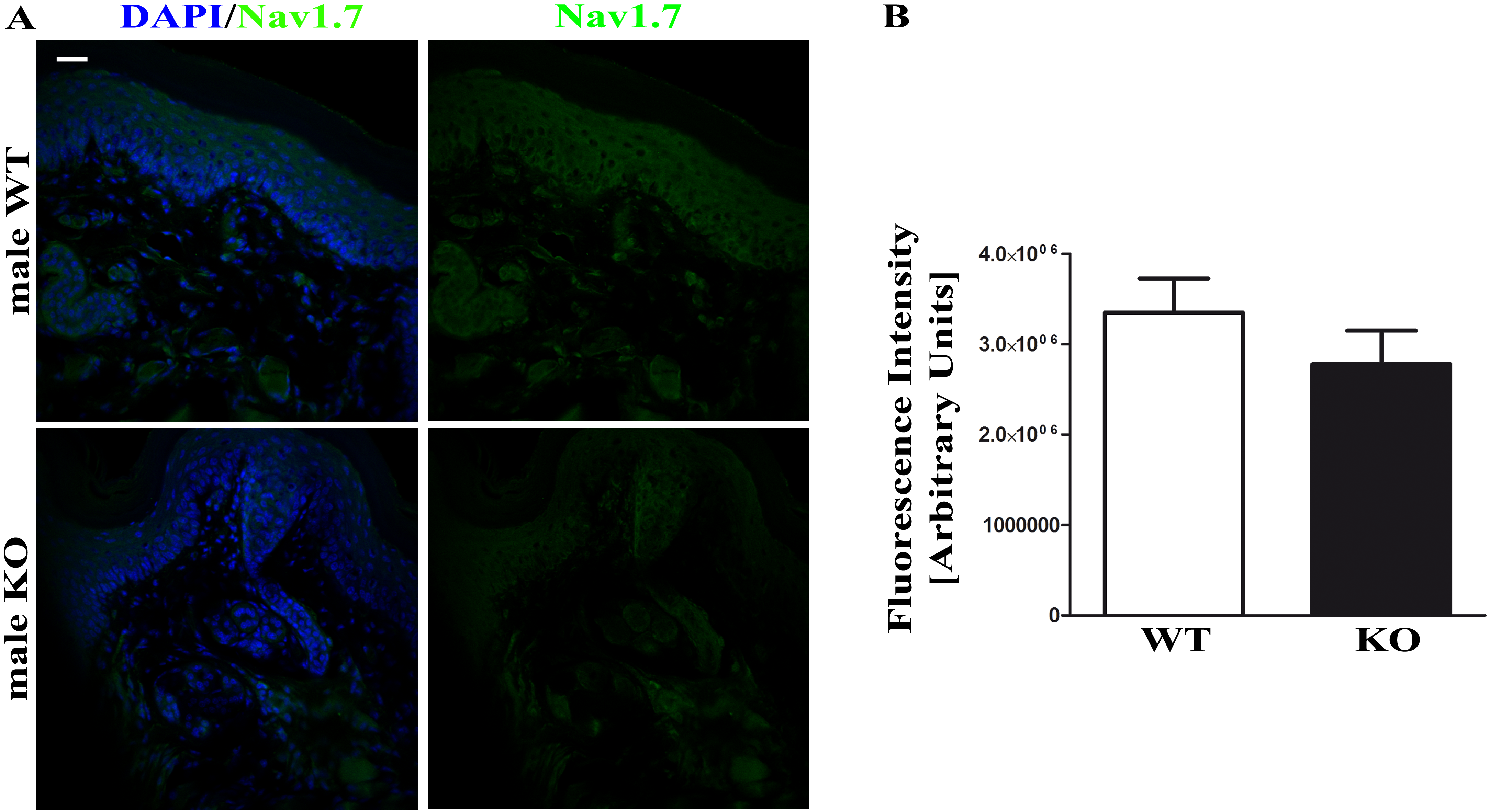

Supplement: Figure S2 — The detection and evaluation of Nav1.7 expression in α-GalA KO males frontal paws. Immunohistochemistry of 12 µm frozen coronal sections of α-GalA KO males (n = 3) showed similar expression at the protein level of the Nav1.7 pain receptor (green) in comparison to their WT controls (n = 3) (A). Scale bar represents 100 µm. Graphical interpretation of Nav1.7 fluorescence quantification (p = 0.1459) (B). Graphical data are expressed as mean±SEM. (TIF) [file pone.0108641.s002.tif]

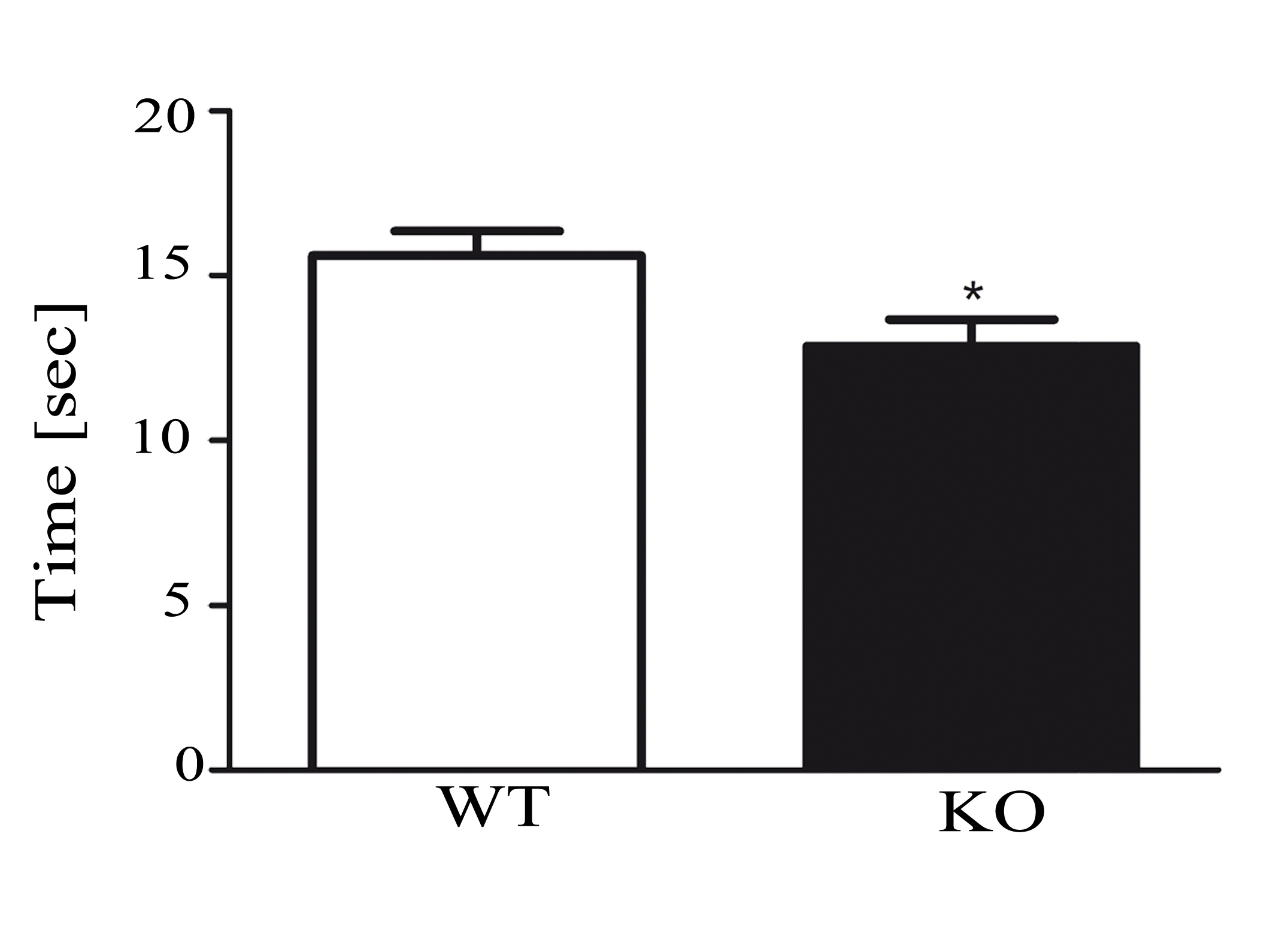

Supplement: Figure S3 — The hot-plate results after 8 months age of α-GalA KO males. Basal sensitivity to noxious temperature stimuli in males of 8 months α-GalA KO (n = 34) and relative WT (n = 23), p<0.0001 as measured with the hot plate (at 52°C). Data are expressed as mean±SEM. (TIF) [file pone.0108641.s003.tif]

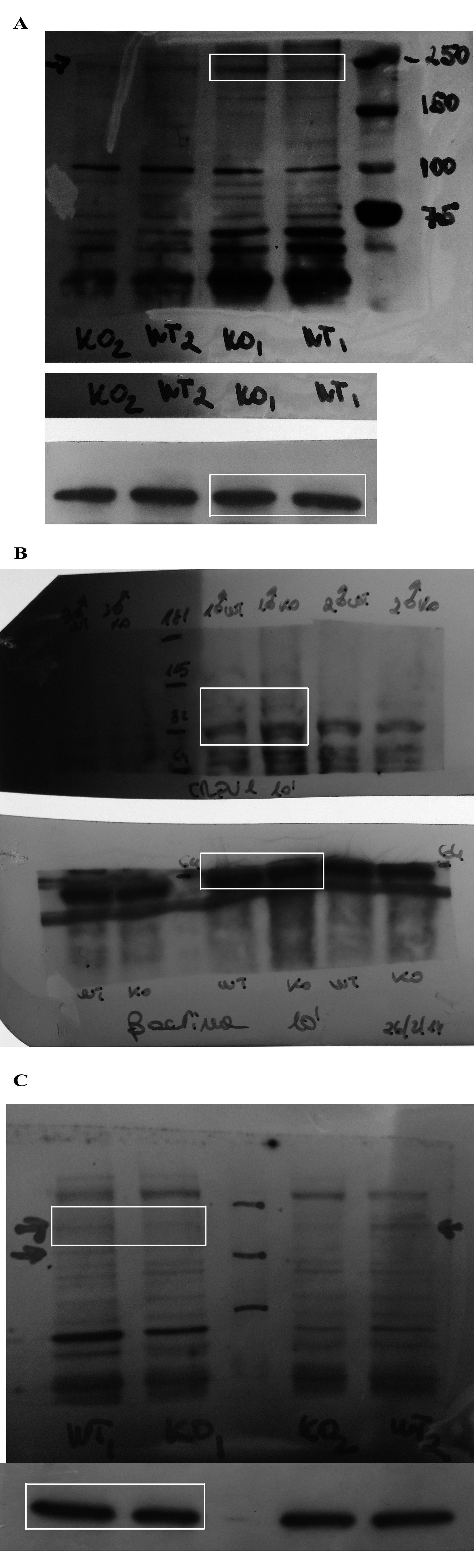

Supplement: Figure S4 — Original WB images. To detect the Nav1.8 (∼220 kDa) protein expression were used 80 µg of tissue lysates separated by 7.5% SDS-polyacrylamide gel (A), for TRPV1 (∼92 kDa) protein expression 40 µg of tissue lysates were separated by 10% SDS-polyacrylamide gel (B), and for TRPM8 (∼127 kDa) protein expression were used 40 µg of tissue lysates separated by 10% SDS-polyacrylamide gel (C). After transfer the membranes were blocked for 1 hour at room temperature and incubated with primary antibodies against specific ionic channels Nav1.8 (1∶200, Santa Cruz), TRPV1 (1∶500, Immunological Sciences) and TRPM8 (1∶200; Santa Cruz) and β-actin (1∶200, Sigma) in 1% BSA in PBST overnight at 4°C. The membranes were rinsed 3 times with PBST, each for 15 minutes and secondary antibodies Horseradish peroxidase-coupled secondary anti-rabbit (1∶1000, Santa Cruz) for TRPV1, TRPM8 and β-actin and secondary anti-goat (1∶5000, Sigma) for Nav1.8 were employed for incubation in 1% BSA rinsed in PBST for 2 hours at room temperature. After washout of secondary-HRP binding antibody membrane was incubated with chemiluminescence substrate (Santa Cruz) for 5 minutes; protein bands were visualized on X-ray (Thermo Scientific). (TIF) [file pone.0108641.s004.tif]
